# Educational Interventions for Nursing Students to Develop Communication Skills with Patients: A Systematic Review

**DOI:** 10.3390/ijerph17072241

**Published:** 2020-03-26

**Authors:** Lorena Gutiérrez-Puertas, Verónica V. Márquez-Hernández, Vanesa Gutiérrez-Puertas, Genoveva Granados-Gámez, Gabriel Aguilera-Manrique

**Affiliations:** Department of Nursing, Physiotherapy and Medicine, Research Group for Health Sciences CTS-451, Universidad de Almeria, 04120 Almeria, Spain; lgp524@ual.es (L.G.-P.); vgp919@ual.es (V.G.-P.); gaguiler@ual.es (G.A.-M.)

**Keywords:** nurse-patient communication, nursing students, patient-centered communication, systematic review, therapeutic communication, training

## Abstract

Introduction: Nursing students establish therapeutic relationships with their patients and as future nursing professionals, they should be trained to be effective communicators. The objective of this systematic review was to know the impact of educational interventions on nursing students to develop their communication skills with patients. Methods: A systematic review of literature was carried out. The following databases were consulted: CINAHL, PubMed (Ovid Medline), SCOPUS and Web of Science. The Preferred Reporting Items for Systematic Reviews and Meta-Analyses guided this review. As for inclusion criteria, published articles in English from 2000 to 2020 were included. The methodological rigor of the included articles was evaluated with the JBI Critical Appraisal Checklist for Randomized Controlled Trial or Quasi-Experimental Studies. Changes in communication skills with the patient after the implementation of an intervention were analyzed. Results: Of the included studies in this systematic review (N = 19), two studies were randomized controlled trials, others were single group quasi-experimental studies (N = 11) and two group quasi-experimental studies (n = 6). The majority of the studies were carried out in the USA (n = 7). The most frequent educational intervention was simulation (n = 11). As for the improvement of communication skills, 13 of the 19 articles found statistically significant differences in patient-centered communication skills of nursing students. Conclusions: This systematic review provides preliminary evidence of the effectiveness of interventions used to train nursing students in patient-centered communication. Although all the interventions obtained significant results in communication skills, it has not yet been determined which methodology is more effective.

## 1. Introduction

Therapeutic communication (TC) is defined as the process of using verbal and nonverbal communication to connect with patients [1]. TC is holistic, patient-centered and involves aspects of the physiological, psychological, environmental and spiritual care of the patient [2]. It is based on understanding and addressing the patient’s situation, including life circumstances, beliefs, perspectives, relevant concerns and needs in order to plan adequate patient care [3,4]. TC between the nurse and patient is considered one of the most significant clinical methods of communication and the basis of nursing care [5]. 

The TC that nurses establish with their patients has been explored in various clinical areas. In particular, with psychiatric patients, it has shown improved health outcomes and decreased clinical anxiety and depression [6]. In the case of chronically ill patients, it has contributed to an increase in adherence to treatment [7], and therefore, an improvement in the self-management of their disease [8]. Additionally, in palliative care, communication skills are essential to provide the patient with effective symptom management, psychosocial and spiritual support and advance care planning [9]. 

A nurse’s ability to communicate in an effective way is essential for developing therapeutic relationships with their patients and obtain greater patient satisfaction. It also minimizes treatment errors and improves the quality of nursing care [10,11]. Patients and their caregivers positively value professionals who attentively listen, the depth of conversation, the moment of delivering information and communicating in an empathetic way [12]. However, various studies have shown that nurses lack communication skills due to inadequate training or a failure to appreciate the importance of patient-centered communication [7,13]. For this reason, nursing professors must find active and effective ways to foster communication skills in the education of nursing students [14].

Teaching and training how to effectively communicate can be complicated due to the variety of potentially difficult conversations that nursing students may face in clinical settings [15]. This situation constitutes a challenge for university professors in charge of educating nursing students [15,16]. Nursing students establish therapeutic relationships with their patients, and as future nursing professionals, they should be trained to be effective communicators [17]. Therefore, the concept of TC should be emphasized in the nursing curriculum to meet the educational needs of the students, as well as the needs of the patients [18,19,20]. However, most interventions aimed at improving patient-centered communication have been for doctors [21,22]. Given the above, the question was posed as to whether nursing students that receive educational interventions could improve their communication skills with patients. Therefore, the objective of this systematic review was to know the impact of educational interventions on nursing students to develop their communication skills with patients. 

## 2. Method

### 2.1. Search Strategy

A systematic review was carried out from September 2019 to January 2020. For this purpose, a critical evaluation of all related evidence was conducted, following a widely documented methodology, in order to answer the specific research question [23]. The Preferred Reporting Items for Systematic Reviews and Meta-Analyses (PRISMA) checklist was used as a guide for the search and presentation of the results [24,25,26,27,28,29,30,31,32,33,34,35,36,37,38,39,40,41,42,43,44,45,46].

An initial search was conducted to obtain information on the breadth of publications and identify the words contained in the titles and abstracts on Google Scholar. A search was then made in the PubMed database (see Box 1) through the US National Library of Medicine in order to identify the Medical Subjects Headings (MeSH). However, the term MeSH ‘therapeutic communication’ and similar terms found were not linked to any educational subgroup in the search tree. Subsequently, a systematic search was performed using all the keywords identified in the following databases: CINAHL, PubMed (Ovid Medline), SCOPUS and Web of Science.

Box 1 **Example of search strategy conducted in PubMed.**

(((((((("students, nursing"[MeSH Terms] AND prelicensure[Title/Abstract]) OR undergraduate[Title/Abstract]) AND intervention[Title/Abstract]) OR effectiveness[Title/Abstract]) AND therapeutic communication[Title/Abstract]) OR nurse-patient communication[Title/Abstract]) OR patient-centered communication[Title/Abstract]) OR interpersonal communication[Title/Abstract]) AND English[Language]

A systematic strategy was used derived from the terms ‘nursing students’ (population); ‘education’ or ‘teaching’ and ‘therapeutic communication’ (intervention); ‘undergraduate’ or ‘prelicensure’ or ‘university’ (context); and ‘effectiveness’ or ‘impact’ (outcome). Finally, manual searches were conducted in the references lists of recent studies and reviews in search of eligible articles that could have been previously lost. 

As for inclusion criteria, the review included quantitative studies whose design were Randomized Clinical Trials (RCT) and quasi-experimental trials published in English from January 2000 to January 2020. Regarding the participants, studies were included with participants that were undergraduate (or pre-licensure) nursing students, regardless of their age, sex or country of origin. With respect to the types of intervention, the review included studies of TC training or patient-centered training. In regards to the types of measured results, the review considered any objectively measured or self-reported quantitative data reporting on therapeutic communication outcomes. 

### 2.2. Data extraction

**Figure 1:**
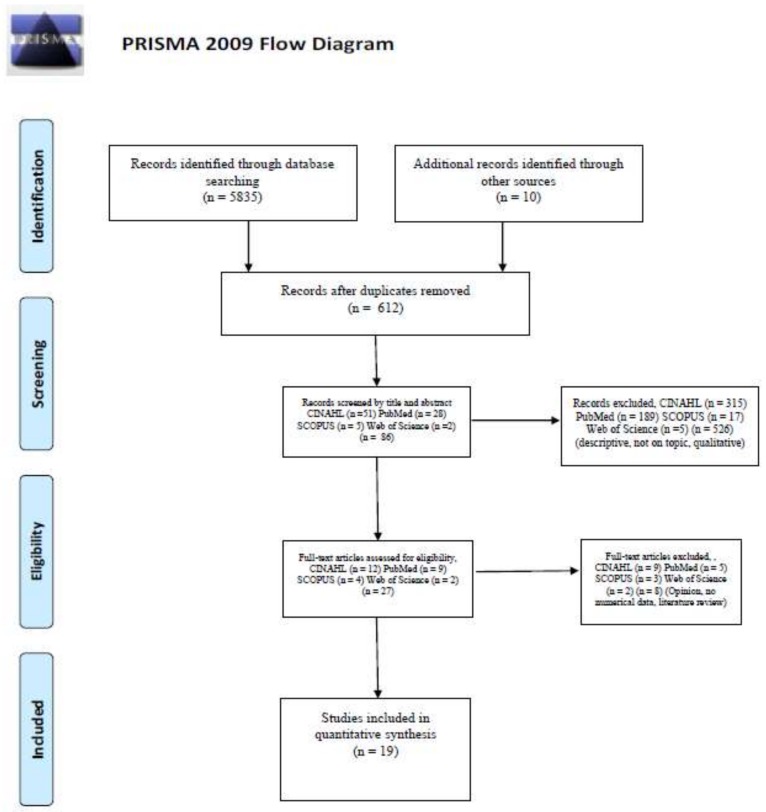
Systematic review flow diagram.

A total of 5,845 articles were identified in the initial search. All citations and abstracts identified in the search strategy were downloaded to Mendeley. The first author (LG) assessed the titles of the articles obtained from the search in the databases. The search yielded 612 articles after eliminating duplicates (see the systematic review flow diagram in Figure 1). The abstracts were reviewed and studies were excluded if: (a) the intervention was not aimed at TC or patient-centered; (b) if the intervention was not aimed at nursing students; (c) articles that were systematic reviews, meta-analysis, qualitative studies, case studies, doctoral thesis or conference abstracts; (d) articles not written in English. Of the 86 abstracts reviewed, 27 articles were selected by the first author for revision of the full text. From the manual search, 10 relevant articles were identified for inclusion in the review. Both reviewers independently analyzed the 27 articles taking the preestablished criteria into account. 

Data from the included articles were reviewed by two independent reviewers (LG and VM), using the JBI-MAStARI data extraction tool. The reviewers extracted information from each of the articles including data on design, theoretical framework, participants, intervention, outcome measures and results. The most relevant characteristics of the studies included in the review are summarized in Table 1. Due to variations in the intervention methods and outcome measures, it was not possible to carry out a meta-analysis. 

### 2.3. Quality appraisal

The selected articles were independently evaluated by two reviewers (GA and VG), before being included in this review. The methodological validity was evaluated using the Joanna Briggs Institute Meta-Analysis of Statistics Assessment and Review Instrument standardized critical appraisal instrument (JBI MAStARI). For the RCTs, the JBI MAStARI for RCTs was used. This checklist is made up of thirteen items. The possible answers to the items are yes, no, unclear or not applicable. If “yes” is answered, a point is obtained. For the study to be included, it had to obtain a score equal to or greater than seven. In the quasi-experimental studies, JBI MASTARI for quasi-experimental studies was used. This checklist is made up of nine items that can be rated yes, no, unclear or not applicable. If “yes” is chosen for the item, a point is obtained. For the study to be included in the systematic review, it had to obtain a score equal to or greater than five. This process enabled an increase in methodological rigor and evaluated possible biases and threats to the validity [25]. The discrepancies between the reviewers of the articles that were to be included in the review were discussed until a consensus was reached. After review, evaluation and discussion, eight articles were excluded that were not based on TC interventions, as defined in the inclusion criteria, or for methodological reasons. Finally, 19 articles were included after confirmation by both reviewers. 

## 3. Results

### 3.1. Characteristics of the study

The overall sample size of the studies included in this review was N = 1,295 participants. In the included studies, there was a great deal of variation in the sample size, ranging from 26 to 147 (median, n = 62). Although the literature search was conducted from the year 2000, the first study included in this review was from 2006. Ninety percent of the studies (n = 18) were carried out in the last ten years and more than fifty percent (n = 10) were conducted in the last five years. Most studies (n = 9) were in mental health. The other areas represented were end-of-life and maternity. As for the study designs, the majority were quasi-experimental studies (n = 17) followed by RCTs (n = 2). In all of the included studies, pre-test and post-test measurements were performed (N = 19) (Table 1).

### 3.2. Theoretical frameworks

Only two of the 19 studies included in this systematic review included a description of a theoretical framework. Donova and Mullen [26] used the Constructivist Learning Theory by Merriam, Cafferella and Baumgartner [27]. While Shorey et al. [28] used a combination of the two frameworks (a) the Self-Efficacy Theory [29]; and (b) the Authentic Learning Concept [30]. 

### 3.3. Intervention characteristics 

Eleven studies used simulation as the intervention. The majority of them (n = 8) used SP to facilitate learning [26,31,32,33,34,35,36,37] and three studies used simulation to aid in the assessment of communication skills, performed by a faculty member [38], peer [39], or both [40]. Nine were carried out through simulation using Standardized Patients (SP) [26,31,32,33,36,37,38,40]. One study used simulation through role playing [39] and with high-fidelity patient (n = 1) [34]. Three studies used innovative educational methodologies, such as a blended learning environment [28], cooperative learning methods [41], and Case-Based learning [42]. Five studies focused their intervention on the development of courses [43,44,45,46,47], and four of the courses indicated the included activities. 

Regarding the contexts of the interventions, nine studies focused on mental health [26,31,33,35,36,37,38,39,40], three studies on end-of-life [32,43,47], one focused on maternity [34], and six studies focused on general patient-centered communication skills [28,41,42,44,45,46]. As for the duration of the interventions, it varied from one hour to one semester (Table 2).

### 3.4. Outcome measures

The included studies reported 19 different instruments of patient-centered communication. Most of the studies provided data on the reliability and validity of the instruments, either in previous studies or calculated for the study they carried out. However, several authors designed the instruments themselves to evaluate interpersonal communication. For six instruments, no validity data was provided [31,32,33,36,38,39]. 

### 3.5. Intervention impact on outcomes

Of the articles included in the review, thirteen determined a statistically significant improvement in the results. More than fifty percent (n = 11) of the studies used simulation as part of the training of patient-centered communication skills. Several studies that used simulation found no statistically significant differences between the groups [33,35,37]. However, they did determine an improvement in the patient-centered communication skills of the intervention group (IG) with respect to the control group (CG) [33,35,37] (see Table 3). 

### 3.6. Quality assessment

Based on JBI criteria for the effectiveness of experimental designs, the two RCTs included were evaluated at level 1C (See Table 1). The quasi-experimental studies (n = 6) with two groups reported a level of evidence of 2C and the experimental studies with a single group (n = 11) reported evidence at level 2D for effectiveness, according to the criteria of evidence levels of JBI [25] (Table 4 and Table 5). 

## 4. Discussion 

This systematic review provides an overview of the research carried out on nursing students in order to develop communication skills with the patient. The objective of this systematic review was to identify, critically evaluate and synthesize the evidence of the impact of educational interventions on nursing students to develop their communication skills with patients. Despite the various educational pedagogies used to develop communication skills with the patient. All research agrees on the importance of developing interventions to improve communication skills with the patient in nursing students. From the main findings, it can be indicated that the majority of the analyzed studies used simulation as the methodology for communication skills training, obtaining statistically significant results. Regarding the countries in which they have carried out the studies, it is worth noting that the majority have been carried out in the USA, these data coincide with those reported in previous research on the analysis of scientific communication publications [48,49]. Regarding theoretical and conceptual frameworks to guide the intervention studies, only two studies used theoretical frameworks to guide the intervention [26,28]. However, the theoretical and conceptual frameworks are essential to develop experimental studies allowing the variables and the relationship between them to be established, described in previous studies. The conceptual frameworks provide information about the subjects, the way of collecting and statistical analysis of the data, making it possible to guide the interventions in the experimental studies and helping the interpretation of the data [50]. 

However, measuring patient-centered communication can be difficult due to the numerous definitions that exist to refer to this type of communication such as TC, nurse–patient communication or interpersonal communication. In addition, there are numerous aspects of communication with the patient that must be considered, as reflected in, for example, the conceptual framework of interpersonal relationships [2]. The instruments used must prove to be valid and reliable. However, only fourteen of the articles reviewed provided data on the validity and reliability of the tool [26,28,34,35,36,37,40,42,43,44,45,46,47]. These issues suggest that researchers should consider the relevance of instruments to assess patient-centered communication before using them. In addition to considering the validity and reliability of the instruments, if they were developed in previous studies, psychometric tests should be performed for the study population. In the case that these instruments were developed by the researchers of the study, they should report the psychometric properties of them. On the other hand, previous studies have indicated the need to develop and validate instruments to assess patient-centered communication skills of nursing students [51,52]. The development of validated instruments to assess communication skills with patients would allow evaluating the impact of the interventions developed on nursing students and determining which interventions are more effective.

Regarding interventions to improve patient-centered communication skills of nursing students, it was observed that the majority focused on simulation, using SP (e.g., [26,32,33]), role-playing [39] or high-fidelity patient [34], to either facilitate learning or evaluate communication skills. Previous studies indicate the importance of incorporating simulation in communication skills training. In particular, simulation provides realistic scenarios that allow nursing students to practice and evaluate TC with patients, without putting real patients at risk [53]. In addition, various studies indicate that the simulation with SP offers nursing students the opportunity to practice TC before clinical practices, being able to improve communication with the patient in the clinical setting [54,55]. Simulation using SP can be effective in teaching patient-centered clinical skills [53,56]. On the other hand, previous studies have shown the training of individuals to treat patients in realistic situations provides an opportunity to improve the competencies of nursing students through human interaction and feedback [57,58]. In this review, all of the studies that implemented simulation conducted feedback with the nursing students. The process of providing feedback during or after the simulation sessions allowed them to address their strengths and weaknesses in order to improve their performance [57,58]. In conclusion, previous research indicates that the implementation of simulation in clinical skills training programs could be useful to improve nurses’ communication skills and the ability to interact with patients [6,52]. In relation to the application of innovative educational pedagogies, various studies have indicated the need to implement new active learning pedagogies that involve students in their training in order to improve their clinical skills [59,60]. Regarding the use of courses as an intervention to improve communication skills with the patient, they have been shown to be effective in some of the included studies. However, the development of courses based on master classes is not recommended as the only educational resource in the training of nursing students and should be combined with other educational pedagogies [61]. In short, previous studies indicate that the new generation of students prefer self-directed, immediate, exciting and immersive experiences [62]. They encourage nurse educators to superimpose the dissonances between traditional teaching and generational learning needs, based on active learning, simulations, reflective learning and educational games [63,64]. Hence, most studies are based on simulation or innovative pedagogies, to encourage student participation in the acquisition of skills.

Following the analysis of the included articles, the contexts were mainly based on mental health [26,31,33,35,36,37,38,39,40], end-of-life [32,43,47] and maternity [34]. Six studies focused on general patient-centered communication skills [28,41,42,44,45,46]. Previous studies have indicated that interventions to teach nurse-patient communication skills target the most challenging clinical interactions [52]. These data are consistent with the studies included in this review. In particular, previous studies indicate that nursing students feel challenged and anxious when they have to talk and interact with mental health patients [65,66]; hence, it is one of the most predominant clinical areas in this review. In addition a review indicated that further studies are needed to improve the available evidence on the clinical practice of nursing students with mental health patients [67]. However, the communication skills involved in everyday conversation with patients are equally important, especially given the perception that nurses lack the time to communicate with patients [68] or with chronically ill patients [7,8], and this was not addressed in the nursing students. Regarding the year that the nursing students were enrolled in, statistically significant improvements were observed in both the students enrolled in their first year [28,32,34,35,42] and those in their last year of the nursing degree [33,37,38,39,44,45,46]. In addition, several studies indicate that communication skills training should be incorporated into the nursing degree curriculum every year. Therefore, the students learn and practice various communication skills before beginning their clinical practices in different clinical areas [15,68]. 

In this systematic review, in which 19 quantitative studies on patient-centered communication interventions in nursing students were reviewed, half of them were found, specifically thirteen [26,28,32,34,36,38,39,40,42,44,45,46,47], to be effective in improving patient-centered communication skills. The differences between the obtained results may be due to the study design, as there was a lot of variability in the designs, sampling, teaching interventions, duration and sample size. In addition, several studies indicated improvements in patient-centered communication skills, although the results were not statistically significant in some of them (e.g., [33,35,37]). On the other hand, only one study evaluated the long-term impact of intervention on nursing students, finding statistically significant differences [38]. It would be necessary for future studies to evaluate the long-term impact of the interventions in order to improve their communication skills with patients and to determine the effectiveness over time. 

A longitudinal study design is recommended to assess the stability and long-term influence of the improvements in communication skills observed in this study. Specifically, observational studies are needed to assess student performance in clinical areas. 

### Strengths and limitations

This review includes the following limitations. First, articles that were not in English were excluded, which may constitute a bias by not considering other languages. Most of the included studies used self-report measures to identify the results and few studies used more objective measures. Furthermore, the heterogeneity of the intervention methods and measurements of the studies’ results prevented a synthesis of results through meta-analysis. The studies need more evidence to address the possibility of bias due to the use of self-report measures and other potential forms of bias [69]. For example, the inclusion of quasi-experimental studies without randomization presents a selection bias. This aspect tends to overestimate the effects of intervention, even though a rigorous methodology and relevant data are presented. The studies of a single group that evaluate the impact of intervention, based on differences between pre-test and post-test measures, can interfere with internal validity by not being compared with students of the same cohort who were not exposed to the intervention. The studies where training was carried out over a period of time can lead to biases, as it is not possible to prove whether changes were due to the intervention or other academic activities. Another risk of bias in the studies is that the instructors who performed the interventions were not blinded due to the nature of the educational intervention studies. On the other hand, it is also important to consider that almost 50% of the studies were carried out within the context of mental health, as communication is a key element of the nurse-patient relationship in this area. However, it would be interesting in future research to be able to deepen the analysis of communication skills learning within the nursing curriculum and the subjects or areas in which it is involved. 

This systematic review had some strengths. First, a broad search was performed using MeSH terms and keywords that addressed the communication of nursing students with the patient; and this search was performed in multiple databases. Despite this, research methods have been systematically applied in this review following the guidelines established for systematic review. In addition, a two-person review of what studies to include and the assessment of their quality increased the rigor of the findings. Therefore, the results obtained expand and update what was known thus far about patient-centered communication interventions for nursing students.

More research is needed to develop instruments that evaluate all aspects that interfere with patient-centered communication in order to improve patient-centered communication skills of nursing students through more effective educational strategies, guided by theoretical frameworks, in a more consistent way. In addition, studies should be carried out that report the perspective of the patient in regards to communication established with the nursing students. 

## 5. Conclusions

This systematic review provides preliminary evidence of the effectiveness of interventions used to train nursing students in patient-centered communication. Although all the interventions obtained significant results in communication skills, it has not yet been determined which methodology is more effective. The majority of the analyzed studies used simulation as the methodology for communication skills training, obtaining statistically significant results. This methodology could be combined with other educational strategies that have indicated improved communication of nursing students with their patients. 

## Figures and Tables

**Table 1:** Main characteristics of the selected studies.

Order Number	1st Author, Date (Country)	MAStARI	Participants	Objetives	Study Design
1	Becker et al. 2006 [31] (USA) 1C	10	n = 147 nursing students enrolled in a psychiatric nursing course (IG = 58; CG = 89).	To evaluate knowledge of depression and therapeutic communication skills SP.	Desing: randomized control group. **Data collection:** pre-test, post-test.
2	Baghcheghi et al. 2011 [41] (Iran) 2C	7	N = 34 sophomore nursing students (16 IG; 18 CG).	To evaluate the effect of tradicional learning and cooperative learning methods on nursing students´communication with patients.	Design: Experimental, observer-blinder two groups study. Data collection: pre-test, post-test.
3	Kim et al. 2012 [34] (Korea) 2C	7	n = 70 sophomores nursing students enrolled in a theoretical course in maternity.	To determine the effect of simulation-based education on the communication skill and clinical competence of nursing students in maternity nursing practicum.	Design: quasi-experimental study, two gropup study. Data Collection: pre-test, post-test.
4	Wittenberg-Lyles et al. 2012 [47] (USA) 2D	7	n = 32 nursing students.	To assess the effects of communication training for the practical nurse.	Design: quasi-experimental pilot study. Data collection: pre-test, post-test.
5	Jo and An 2013 [43] (Korea) 2C	7	n = 39 nursing students (19 IG; 20 GC) from two universities.	To examine the effects of a humanistic end-of-life care course on South Korean undergraduate nursing students’ attitudes toward death, death anxiety, and communication skills.	Design: quasi-experimental two group study. Data collection: Pre-test, post-test.
6	Lau and Wang 2013 [44] (China) 2D	7	n = 62 fourth-year nursing students enrolled CST course.	To develop a learner-centered Communication Skills Training (CST) course; (2) to evaluate the course by comparing scores for communication skills, clinical interaction, interpersonal dysfunction, and social problem-solving ability.	Design: quasi-experimental single group study, two-phase mixed methods Data collection: pre-test, post-test.
7	Lin et al. 2013 [35] (Taiwan) 1C	9	n = 26 first year nursing students (14 IG; 12 CG).	To examine the effectiveness of using SP with SP feedback and group discussion to teach Interpersonal and communication skills (IPCS) in nursing education.	Desing: Randomized Controlled Study two group. Data collection: pre-tets, post-test.
8	Lau and Wang 2014 [45] (China) 2D	7	n = 59 fourth-year nursing students attended the summer camp program.	To develop a learner-centered educational summer camp program for nursing students and to evaluate the effectiveness of the camp program on enhancing the participants’ communication skills.	Design: quasi-experimental single group study, two-phase mixed methods. Data collection: pre-test, post-test.
9	Webster 2014 [38] (USA) 2D	7	n = 89 senior baccalaureate nursing students enrolled in a psychiatric clinical course.	To determine the effectiveness of SPEs as a teaching modality to improve nursing students’ use of therapeutic communication skills with individuals with mental illness.	Design: quasi-experimental, one group study. Data Collection: pre-test, post-test.
10	Bloomfield et al. 2015 [32] (UK) 2D	6	n = 28 second-year nursing students and fourth-year medical students from a population of N = 180 nursing students and N = 450 medical students.	To design, implement, and evaluate an educational intervention employing simulated patient actors to enhance students’ abilities to communicate with dying patients and their families.	Design: quasi-experimental single group study, two-phase mixed methods. Data Collection: pre-test, post-test.
11	Yoo and Park 2015 [42] (Korea) 2C	7	n = 143 (72 IG; 71 CG) sophomore undergraduate nursing student enrolled in a mandatory health communication course from a population of N = 151.	To evaluate the effectiveness of Case-based learning on undergraduate nursing students in the health communication course.	Design: quasi-experimental two group study. Data collection: pre-test, post-test.
12	Lai 2016 [40] (Taiwan) 2D	7	n = 50 quasi-experimental single group study.	To implement an online video peer assessment system to scaffold their communication skills and to examine the effects and validity of the peer assessment.	Desing: quasi-experimental single group study. Data collection: pre-test, post-test.
13	Martin and Chanda 2016 [36] (USA) 2D	8	n = 28 prelicensure nursing students enrolled in a mental health nursing theory and clinical course.	To introduce therapeutic communication simulations with emphasis on symptoms related to psychiatric disorders as a part of mental health theory and clinical courses.	Design: quasi-experimental, one group. Data collection: pre-test, post-test.
14	Taghizadeh et al. 2017 [46] (Iran) 2D	8	n = 66 last year nursing students and n = 132 patients.	To determine the impact of teaching communication skills to nurse students on the quality of care given by nursing students.	Design: quasi-experimental single group study. Data collection: pre-test, post- test.
15	Shorey et al. 2018 [28] (China) 2D	8	n = 124 first-year undergraduate nursing students enrolled in the nursing course.	To evaluate the effectiveness of blended learning pedagogy in a redesigned communication module among nursing undergraduates in enhancing their satisfaction levels and attitudes towards learning communication module as well as self-efficacy in communication.	Design: quasi-experimental single group study. Data Collection: pre-test, post-test.
16	Blake and Blake 2019 [39] (USA) 2D	5	n = 32 nursing students in their capstone course from a population of N = 35.	To examine the effects of a nursing lab simulation used to increase the self-efficacy of nursing students with their ability to use effective communication.	Design: quasi-experimental single group. Data collection: pre-test, post-test.
17	Donovan and Mullen 2019 [26] (USA) 2D	7	n = 116 undergraduate nursing students registered for three successive mental health nursing courses during academic year from a population of N = 160 (RR 72.5%).	To examine the efficacy of learned classroom therapeutic communication techniques applied to a standardized patient mental health simulated experience.	Design: quasi-experimental single group study. Data collection: pre-test, post-test.
18	Gaylle 2019 [33] (USA) 2C	7	n = 65 senior students enrolled in a psychiatric clinical rotation at a public university from a population of N = 67 (RR 97%). (IG = 32; CG = 33).	To explored the effects of in-simulation and postsimulation debriefing on students’ knowledge, performance, anxiety, and perceptions of the debriefing process.	Design: quasi-experimental, two group study. Data collection: pre-test, post-test.
19	Ok et al. 2019 [37] (Turkey) 2C	6	n = 85 third-year nursing students enroled in a course on mental health and psychiatric at two different universities from a population of N = 103 (RR 82.5%). (IG = 52; CG = 33)	To measure the impact of using standardized patient simulation (SPS) prior to clinical practice on the anxiety levels and communication skills.	Design: quaxi-experimental two group Data collection: pre-test, post-test

IG, Intervention Group; CG, Control Group; SP, Standardized Patient; CST, Communication Skills Training; IPCS, Interpersonal Communication Skills; SPEs, Standardized Patient Experiences; SPS, Standardized Patient Simulation.

**Table 2:** Intervention characteristics.

Order Number	1st Author, Date (Country)	Participants	Study Design	Theoretical Framework	Intervention	Quantitative Measures
1	Becker et al. 2006 [31] (USA)	n = 147 nursing students enrolled in a psychiatric nursing course (IG = 58; CG = 89).	Design: randomized control group. Data collection: pre-test, post-test.	Not mentioned.	Simulation—using Standardized Patient (SP). Lectures on therapeutic communication and nursing care of clients with depression (both group), Interview SP, debriefing, videotape self-analysis with accompanying handbook. Duration: once a week, 7 weeks. Interview SP (30 min), debriefing (30 min), videotape self-analysis (after 1 week of the SP encounter). CG - usual classroom lecture format.	Students: Communication Knowledge Test (CKT), developed by the authors for this study. Student Self-Evaluation of SP Encounter (SSPE), developed by the authors for this study. Patients: SP checklist, developed by the authors for this study. Standardized Patient Interpersonal Ratings (SPIR), developed by the authors for this study.
2	Baghcheghi et al. 2011 [41] (Iran)	N = 34 sophomore nursing students (16 IG; 18 CG).	Design: Experimental, observer-blinder two groups study. Data collection: pre-test, post-test.	Not mentioned.	Cooperative learning methods. (work in group) Activities included in lectures: Socratic questioning, paired discussion of homework assignments, paired pop quizzes, small group discussion of case scenarios, paired concept-map generation exercises, student identification of examples for concepts being discussed, and think-pair-share exercises. Each group would be responsible for presenting a 15 to 20-minute review of information from their particular content category to the class. Throughout the semester the group members evaluated each other with a weekly evaluation tool; feedback. Duration: one semester. CG—usual classroom lecture format.	Nursing Students’ communication with patient scale.
3	Kim et al. 2012 [34] (Korea)	n = 70 sophomores nursing students enrolled in a theoretical course in maternity.	Design: quasi-experimental study, two group study. Data Collection: pre-test, post-test.	Not mentioned	Simulation—using high-fidelity patient simulator. Duration: 9 h over three weeks (briefing, simulation lab, debriefing). CG—usual classroom lecture format.	Communication Skills Tool. Clinical Competence Tool (CCT).
4	Wittenberg-Lyles et al. 2012 [47] (USA)	n = 32 nursing students.	Design: quasi-experimental pilot study. Data collection: pre-test, post-test.	Not mentioned	COMFORT communication and consulting course. interactive, educational training session and taught students using a combination of PowerPoint lectures, case studies, small group discussions, and exercises. Students were exposed to concepts including narrative clinical practice, person-centered messages, the task and relational components in all interactions, and participated in 3 encounters using these concepts. Duration: 3h.	Course Experience Questionnarie (CEQ) created by authors for this study. Perceived Importance of Medical Communication (PIMC). Communication Skill Attitude Scale (CSAS). Caring Self-Efficacy Scale (CES).
5	Jo and An 2013 [43] (Korea)	n = 39 nursing students (19 IG; 20 GC) from two universities.	Design: quasi-experimental two group study. Data collection: Pre-test, post-test.	Not mentioned.	End-of-life- Care course teaching included uses humanistic educational methods such as lectures, group discussion, watching a movie, analysis of novel and poem, appreciation of music, and collage art, role-play, and sharing personal experiences. Duration: 2h x 16 weeks. CG—usual classroom lecture format.	Attitudes toward death. Death Anxiety Scale (DAS). Communication Assessment Tool (CAT).
6	Lau and Wang 2013 [44] (China)	n = 62 fourth-year nursing students enrolled CST course.	Design: quasi-experimental single group study, two-phase mixed methods Data collection: pre-test, post-test.	Not mentioned.	Communication Skills Training (CST) course. Included theoretical lectures and practical components (Immediate feedback; Role Playing; Group discussion; didactical games). Duration: two day, 8 h per day.	Communication Ability Scale (CAS) Clinical Interaction Scale (CIS). Interpersonal Dysfunction Checklist (IDC). Social Problem Solving Inventory-Revised (C-SPSI-R).
7	Lin et al. 2013 [35] (Taiwan)	n = 26 first year nursing students (14 IG; 12 CG).	Design: Randomized Controlled Study two group. Data collection: pre-test, post-test.	Not mentioned.	Simulation - using SP. Briefing; scenario demonstration; role-playing. Duration: 2-day (SP assessments with SP feedback and group discussion). CG—usual classroom lecture format.	Interpersonal Communication Skills (IPCS) assessment tool. Student Learning Satisfaction (SLS) Scale.
8	Lau and Wang 2014 [45] (China)	n = 59 fourth-year nursing students attended the summer camp program.	Design: quasi-experimental single group study, two-phase mixed methods. Data collection: pre-test, post-test.	Not mentioned	Educational Summer Camp Program on Communication Skills—three sharing sessions and five experimental learning games. Sharing sessions on self-exploration, teambuilding, and clinical interaction. Experiential learning games were used as learning strategies (icebreaker, self-discovery, team building, problem solving, and communication). Duration: 3 days	Communication Ability Scale (CAS) Clinical Interaction Scale (CIS). Interpersonal Dysfunction Checklist validated Chinease (IDC). Social Problem Solving Inventory-Revised (SPSI-R).
9	Webster 2014 [38] (USA)	n = 89 senior baccalaureate nursing students enrolled in a psychiatric clinical course.	Design: quasi-experimental, one group study. Data Collection: pre-test, post-test.	Not mecioned.	Simulation—using SP, simulations were video-recorded, watched their video and conducted a self-reflection of strengths and areas for improvement; debriefing conducted by faculty using a problem-based learning approach. Duration: Two SPEs, one at the beginning of the semester and one at the end of the semester. 15–20 min sessions.	The effectiveness of the use of SPEs to teach therapeutic communication skills in psychiatric nursing ckecklist created by author for this study. Feedback from faculty ckecklist created by author for this study.
10	Bloomfield et al. 2015 [32] (UK)	n = 28 second-year nursing students and fourth-year medical students from a population of N = 180 nursing students and N = 450 medical students.	Design: quasi-experimental single group study, two-phase mixed methods. Data Collection: pre-test, post-test.	Not mentioned.	Simulation—using SP (two scenarios), pre-briefing; simulation; debrief. Duration: 45 min including pre-brief, simulation and debrief.	students’ perceived levels of confidence, competence, and concern when communicate with dying patients and their families questionnaire created by authors for this study.
11	Yoo and Park 2015 [42] (Korea)	n = 143 (72 IG; 71 CG) sophomore undergraduate nursing student enrolled in a mandatory health communication course from a population of N = 151.	Design: quasi-experimental two group study. Data collection: pre-test, post-test.	Not mencioned.	Case-Based Learning (CBL) - as teaching activity in a course. Five authentic cases of patient-nurse communication. (Stage of each 5-Cases: Case presentation; Student´s case analysis individually; group discussion and analysis; finding proper solution by group; group presentation of the cases). Duration: 28 h. CG –traditional lecture-based learning.	Communication Assessment Tool (CAT). Problem-Solving Inventory (PSI). Instructional Materials Motivation Scale (IMMS).
12	Lai 2016 [40] (Taiwan)	n = 50 quasi-experimental single group study.	Design: quasi-experimental single group study. Data collection: pre-test, post-test.	Not mentioned.	Simulation—using SP an online video peer assessment system. Recorded therapeutic consultation with a SP and uploaded to YouTube; peer assessment and feedback through a web-based assessment system; expert evaluation (two rounds; different scenarios). Duration: SP twice; once in the mid-term exam week and the other in the final exam week. Duration not stated.	Interpersonal Communication Assessment Scale (ICAS).
13	Martin and Chanda 2016 [36] (USA)	n = 28 prelicensure nursing students enrolled in a mental health nursing theory and clinical course.	Design: quasi-experimental, one group. Data collection: pre-test, post-test.	Not mentioned.	Simulation using SP (three stations; two simulation sessions). Briefing; simulation with two standardized patients and a case study; debriefing. Duration: 40-50 min simulation followed by an hour debriefing.	Confidence with Communication Skill Scale. Therapeutic communication and nontherapeutic communication techniques, checklist created by authors, with the purpose of evaluating skills that would occur during the SP encounters.
14	Taghizadeh et al. 2017 [46] (Iran)	n = 66 last year nursing students and n = 132 patients.	Design: quasi-experimental single group study. Data collection: pre-test, post- test.	Not mentioned.	Communication Training Course. lectures and workshops using educational equipment and technology. Duration: 6 h.	Student´s Communication skills checklist created by the authors for this study. Quality of Care Questionnaire for Patients.
15	Shorey et al. 2018 [28] (China)	n = 124 first-year undergraduate nursing students enrolled in the nursing course.	Design: quasi-experimental single group study. Data Collection: pre-test, post-test.	Bandura´s self-efficacy theory (1997).	Blended learning environment face-to-face each week for tutorials (Role-playing and problem-based learning); lecture materials online (breeze presentations, PowerPoints slides, and multi-media components, delivered) online quizzes, discussion forums, and reflection exercises; assessment (analyzing real life clinical scenarios by creating online videos; interview with SP). Duration: 13 weeks. Four modular credit x 10 h (2–3 h for face-to-face tutorial or lecture and 7–8 h for the self-directed learning).	Blended Learning Satisfaction Scale (BLSS). Communication Skills Attitude Scale (CSAS). Communication Skills subscale of the Nursing Students Self-Efficacy Scale (C-NSSES).
16	Blake and Blake 2019 [39] (USA)	n = 32 nursing students in their capstone course from a population of N = 35.	Design: quasi-experimental single group. Data collection: pre-test, post-test.	Not mentioned.	Simulation—role-playing, debriefing Duration: a week.	Self-efficacy related to therapeutic communication, developed by the authors for this study. A rubric for therapeutic and nontherapeutic statements or actions developed by the authors for this study.
17	Donovan and Mullen 2019 [26] (USA)	n = 116 undergraduate nursing students registered for three successive mental health nursing courses during academic year from a population of N = 160 (RR 72.5%).	Design: quasi-experimental single group study. Data collection: pre-test, post-test.	Constructivist learning theory (Merriam et al. 2012).	Simulation—using SP. Lectures on therapeutic communication techniques, which included readings, video clips with discussion; simulation; debriefing. Duration: 60 min including briefing, simulation and debriefing.	Confidence Simulation, with a dimension about level of confidence of learned therapeutic communication skills.
18	Gaylle 2019 [33] (USA)	n = 65 senior students enrolled in a psychiatric clinical rotation at a public university from a population of N = 67 (RR 97%). (IG = 32; CG = 33).	Design: quasi-experimental, two group study. Data collection: pre-test, post-test.	Not mentioned.	Simulation—using SP (four scenarios) briefing; simulation; In simulation-debriefing. Duration: one week. CG - briefing, simulation, postsimulation debriefing.	Students’ knowledge of psychiatric assessment. Therapeutic communication checklist created by author. Students’ perceived anxiety related to a psychiatric clinical practicum created by author. Perceptions of the debriefing experience checklist created by author for this study.
19	Ok et al. 2019 [37] (Turkey)	n = 85 third-year nursing students enrolled in a course on mental health and psychiatric at two different universities from a population of N = 103 (RR 82.5%). (IG = 52; CG = 33)	Design: quasi-experimental two group Data collection: pre-test, post-test	Not mentioned.	Simulation—using SP theoretical lecture on communication skills and schizophrenia; simulation using SP, debriefing. Duration: 5 hours theoretical lectures, 10–12 min simulation, 30–35 min debriefing. CG—Theoretical lectures and clinical practices.	Communicational Skills Inventory (CSI) State-Trait Anxiety Inventory (STAI)

IG, Intervention Group; CG, Control Group; SP, Standardized Patient; CKT, Communication Knowledge Test; SSPE, Student Self-Evaluation of Standardized Patient Encounter; SPIR, Standardized Patient Interpersonal Ratings; CST, Communication Skills Training; CCT, Clinical Competence Tool; CEQ, Course Experience Questionnarie; PIMC, Perceived Importance of Medical Communication; CSAS, Communication Skill Attitude Scale; CES, Caring Self-Efficacy Scale; DAS, Death Anxiety Scale; CAT, Communication Assessment Tool; CAS, Communication Ability Scale; CIS, Clinical Interaction Scale; IDC, Interpersonal Dysfunction Checklist; C-SPSI-R, Social Problem Solving Inventory Revised; IPCS, Interpersonal Communication Skills; SLS, Student Learning Satisfaction; SPEs, Standardized Patient Experiences; CBL, Case-Based Learning; PSI, Problem-Solving Inventory; IMMS, Instructional Materials Motivation Scale; ICAS, Interpersonal Communication Assessment Scale; BLSS, Blended Learning Satisfaction Scale; C-NSSES, Communication Skills subscale of the Nursing Students Self-Efficacy Scale; CSI, Communicational Skills Inventory; STAI, State-Trait Anxiety Inventory.

**Table 3:** Main results and conclusions.

Order Number	1st Author, Date (Country)	Findings	Conclusions
1	Becker et al. 2006 [31] (USA)	No significant differences were found between the two groups on measures of interpersonal skills, therapeutic communication skills, and knowledge of depression.	Further research is needed, this study support the use of SPs in nursing education for communication skills training.
2	Baghcheghi et al. 2011 [41] (Iran)	The results showed that no significant difference between the two groups in students’ communication skills scores before the teaching intervention (p > 0.05), but did show a significant difference between the two groups in the interaction skills and problem follow up sub-scales scores after the teaching intervention (p < 0.05).	This study provides evidence that cooperative learning is an effective method for improving and increasing communication skills of nursing students especially in interactive skills and follow up the problems sub-scale, thereby it is recommended to increase nursing students’ participation in arguments by applying active teaching methods which can provide the opportunity for increased communication skills.
3	Kim et al. 2012 [34] (Korea)	The communication skill score of the experimental group that participated in simulation-based education increased 0.58 points and the control group increased 0.09 points, indicating a significant difference between the two groups (p = 0.020). The clinical competence score of the experimental group that participated in simulation-based education increased 0.63 points, and the score for the control group increased 0.15 points, indicating a significant difference between the two groups (p = 0.009).	Simulation-based education in maternity is effective in promoting communication skill and clinical competence.
4	Wittenberg-Lyles et al. 2012 [47] (USA)	The practical nurses’ exposure to the COMFORT communication training allowed students to see its benefits, resulting in more positive attitudes to communication skills learning as measured by the CSAS (p < 0.000). The COMFORT communication curriculum also increased perceptions of the importance of communication in nurse training as assessed by the PIMC (p < 0.009). In addition, COMFORT training resulted in an increase in practical nurses’ reported self-efficacy in using communication skills with patients and families, although no statistically differences were found (p = 0.052).	This study shows promise for the feasibility and use of the CONFORT curriculum for nursing students communication training.
5	Jo and An 2013 [43] (Korea)	Attitudes toward death (p = 0.027) and communication skills (p = 0.008) appeared to have significantly increased in the experimental group. However, death anxiety (p = 0.984) did not significantly differ between the two groups after intervention.	The course is effective in reducing negative attitudes toward death and increasing the communication skills of nursing students.
6	Lau and Wang 2013 [44] (China)	There were significantly increase between students: the mean pre-test and post-test scores for communication ability (p = 0.015). there were improvement in the scores for content of communication and handling of communication barriers (p < 0.001). In addition, the training was practically important, as indicated by the effect size of 2.39 in the score for the handling of communication barriers. Although the scales of communication ability, clinical interaction, interpersonal dysfunction, and social problem solving were improved, they were not statistically significant (p >.05).	The course was effective in improving communication skills in nursing students.
7	Lin et al. 2013 [35] (Taiwan)	All participants expressed high SLS (94.44%) and showed significant (p ≤ 0.025) improvements on IPCS total scores, interviewing, and counseling. However, there were no significant differences between groups (p = 0.374).	Using SPs to teach IPCS to nursing students produced a high SLS, but future studies are needed to confirm the effectiveness of SP feedback and group discussions.
8	Lau and Wang 2014 [45] (China)	The analysis showed a significant difference between the mean pretest and posttest scores of the subscales (p = 0.003) and total communication skills scores (p < 0.0001). There was a statistically significant increase in the cognition of communication scores from pre-test to post-test (p < 0.0001), content of communication (p = 0.009), and handling of communication barriers (p < 0.001). The mean pretest and posttest CIS total scores increased (p < 0.0001), sympathetic consideration (p < 0.0001), active listening (p = 0.001), and taking the initiative in care subscales (p = 0.009). The scores of positive problem orientation subscale of the SPSI-R improved (p = 0.037).	The Educational Summer Camp Program was effective in improving nursing students´communication skills.
9	Webster 2014 [38] (USA)	The students did not demonstrate significant improvement on 2 of the 14 evaluation criteria -approaching client with a nonthreatening body stance (p = 0.218) and introducing self (p = 0.74)- although there was improvement noted for the two evaluation criteria. There was improvement noted in anxiety, students’ ability to establish eye contact, to engage in efforts to put the patient at ease, safety assessments, the ability to set limits on inappropriate behavior (p < 0.05). In building a therapeutic relationship, Improvements were also noted in all three of these areas (using therapeutic communication techniques; responding appropriately to verbal statements and responding appropriately to nonverbal behavior), (p < 0.05). The ability to validate the meaning of a patient’s response increased significantly. Last, the appropriate termination were increase significantly for these two areas (summarizing content of interaction, terminating appropriately), (p < 0.05).	This study suggests that the use of SPEs is an effective methodology for promoting therapeutic communication skills in nursing students.
10	Bloomfield et al. 2015 [32] (UK)	After the simulation, self-perceived confidence levels when communicating with the family and friends of dying patients increased significantly (p < 0.05). The majority of students reported increased levels of competence when talking with the family of dying patients (p < 0.05).	Simulation was found to be an effective means of preparing nursing students to communicate with dying patients and their families.
11	Yoo and Park 2015 [42] (Korea)	A significant increase in the communication skills score of the intervention group was observed (p < 0.001) while a slight increase was observed for the control group (p < 0.001). There was a significant difference in the communication skills of the two groups (p < 0.001). A significant decrease in the problem solving ability score of the intervention group was observed (p < 0.001), whereas an increase was observed in the control group (p < 0.001). A significant improvement was observed for the problem-solving ability of the intervention group, as compared to the control group (p < 0.001). Finally, scores for learning motivation showed a significant increase (p < 0.001), for the intervention group, whereas a decrease (p > 0.05), was observed for the control group. Moreover, a significant difference was found in the learning motivation scores of the two groups (p < 0.001).	This finding suggests that case-based learning is an effective learning and teaching method.
12	Lai 2016 [40] (Taiwan)	The scores given by the peers were significatly corelated with those given by experts (r = 0.36, p<0.05). In relation, students’ attitudes toward the peer assessment activities. Overall, the mean scores of each item were greater than 4 (agree) which means the students were satisfied with the peer assessment learning activities.	The nursing students had improved their skills in therapeutic communication as a result of the networking peer assessment. Expert evaluation scores showed that students’ communication performance, when involved in peer assessments, significantly improved.
13	Martin and Chanda 2016 [36] (USA)	There was significant improvement (p = 0.000), in student’s self-reported confidence with their communication skills and knowledge following a mental health simulation experience using standardized patients.	A therapeutic communication mental health simulation give before students participating in their clinical experience should be integrated into undergraduate nursing education.
14	Taghizadeh et al. 2017 [46] (Iran)	The results showed that there was a significant difference between the mean quality of patients’ care prior to and following the intervention (p≤0.001). Also, there was a significant difference between the means for nursing student’s’ communication skills before and after the intervention (p≤0.001). Moreover, there was a significant correlation between mean scores of students and the quality of care and communication skills (p≤0.001).	The course was effective in improving communication skills in nursing students.
15	Shorey et al. 2018 [28] (China)	There was a statistically significant increase in the BLSS scores from pre-test to post-test (p = 0.012). Similarly, a statistically significant increase in the CSAS scores were seen from pre-test to post-test (p = 0.042). There was also a statistically significant increase in the C-NSSES scores from pre-test to post-test (p = 0.003).	Participants had enhanced satisfaction levels with blended learning pedagogy, better attitudes in learning communication skills, and improved communication self-efficacies at posttest.
16	Blake and Blake 2019 [39] (USA)	An improvement in student self-efficacy in therapeutic communication skills after the course simulation as indicated by the five questions were all significant with p < 0.01.	The lab simulation was helpful in improving students regarding their therapeutic communication skills.
17	Donovan and Mullen 2019 [26] (USA)	The pre/post results suggest the standardized simulated experience enhanced nursing student confidence p < 0.001. These results suggest that the student nurse confidence in therapeutic communication with a mental health patient had increased.	Simulation with SPs promoted an active learning environment that highlighted individualized confidence in therapeutic communication skills through a realistic application process.
18	Gaylle 2019 [33] (USA)	The overall change from pretest to posttest for therapeutic communication for both groups combined was statistically significant and practically important with a large effect size of 1.34 (Cohen d). On average, both groups showed statistically significant improvement (p < 0.05). The in-simulation group demonstrated a greater increase in therapeutic-communication techniques and a larger decrease in nontherapeutic communication than their peers in the post-simulation group. Differences in means between the in-simulation and the post-simulation groups for therapeutic communication (mean, 1.39 and 0.83) but there are not statistically differences significant between groups.	In simulation debriefing is an effective tool for teaching therapeutic communication to nursing students.
19	Ok et al. 2019 [37] (Turkey)	There are differences between the students who received and who did not receive SPS in terms of the scores obtained from the STAI-S (p = 0.01), STAI-T (p = 0.046), but there are not statistically differences in CSI (p = 0.09), except for the subscale cognitive of the CSI (p = 0.043). The comparison of the scores obtained by the intervention group prior to and after the SPS shows a statistically meaningful decrease in the anxiety levels (p = 0.001; p = 0.009) and a statistically meaningful increase in the communication skills of the intervention group after the simulation exercise (p = 0.001), except for the emotional subscale (p = 0.074).	Simulation with SPs may help nursing students gain experience and increase communication skills with patients.

SP, Standardized Patient; SLS, Student Learning Satisfaction; CIS, Clinical Interaction Scale; IPCS, Interpersonal Communication Skills; SPSI-R, Social Problem Solving Inventory Revised; SPEs, Standardized Patient Experiences; BLSS, Blended Learning Satisfaction Scale; CSAS, Communication Skill Attitude; C-NSSES, Communication Skills subscale of the Nursing Students Self-Efficacy Scale; SPS, Standardized Patient Simulation; CSI, Communicational Skills Inventory; STAI, State-Trait Anxiety Inventory

**Table 4:** Results of critical appraisal for quasi-experimental studies.

Order Number	MAStARI Question	Q1	Q2	Q3	Q4	Q5	Q6	Q7	Q8	Q9	Score
	**1st author, date (Country)**										
1	Baghcheghi et al. 2011 [41] (Iran)	Yes	Yes	Unclear	Yes	Yes	No	Yes	Yes	Yes	7
2	Kim et al. 2012 [34] (Korea)	Yes	Unclear	Unclear	Yes	Yes	Yes	Yes	Yes	Yes	7
3	Wittenberg-Lyles et al. 2012 [47] (USA)	Yes	Unclear	Yes	No	Yes	Yes	Yes	Yes	Yes	7
4	Jo and An 2013 [43] (Korea)	Yes	Yes	Unclear	Yes	Yes	Unclear	Yes	Yes	Yes	7
5	Lau and Wang 2013 [44] (China)	Yes	Yes	Yes	No	Yes	Unclear	Yes	Yes	Yes	7
6	Lau and Wang 2014 [45] (China)	Yes	Yes	Yes	No	Yes	Unclear	Yes	Yes	Yes	7
7	Webster 2014 [38] (USA)	Unclear	Yes	Yes	No	Yes	Yes	Yes	Yes	Yes	7
8	Bloomfield et al. 2015 [32] (UK)	Yes	Yes	Yes	No	Yes	Yes	Yes	Unclear	Unclear	6
9	Yoo and Park 2015 [42] (Korea)	Yes	No	Unclear	Yes	Yes	Yes	Yes	Yes	Yes	7
10	Lai 2016 [40] (Taiwan)	Yes	Yes	Yes	No	Yes	Yes	Yes	Yes	No	7
11	Martin and Chanda 2016 [36] (USA)	Yes	Yes	Yes	No	Yes	Yes	Yes	Yes	Yes	8
12	Taghizadeh et al. 2017 [46] (Iran)	Yes	Yes	Yes	No	Yes	Yes	Yes	Yes	Yes	8
13	Shorey et al. 2018 [28] (China)	Yes	Yes	Yes	No	Yes	Yes	Yes	Yes	Yes	8
14	Blake and Blake 2019 [39] (USA)	Yes	Yes	Yes	No	Yes	No	Yes	No	No	5
15	Donovan and Mullen 2019 [26] (USA)	Yes	Yes	Yes	No	Yes	No	Yes	Yes	Yes	7
16	Gaylle 2019 [33] (USA)	Unclear	Yes	No	Yes	Yes	Yes	Yes	Yes	Yes	7
17	Ok et al. 2019 [37] (Turkey)	Yes	No	Unclear	Yes	Yes	Yes	No	Yes	Yes	6

**Table 5:** Results of critical appraisal for Randomized Controlled Trials.

Order Number	MAStARI Question	Q1	Q2	Q3	Q4	Q5	Q6	Q7	Q8	Q9	Q10	Q11	Q12	Q13	Score
	**1st author, date (Country)**														
1	Becker et al. 2006 [31] (USA)	Yes	No	Yes	Yes	No	No	Yes	Yes	Yes	Yes	Yes	Yes	Yes	10
2	Lin et al. 2013 [35] (Taiwan)	Yes	Unclear	Unclear	Yes	Unclear	Yes	Yes	Yes	No	Yes	Yes	Yes	Yes	9
